# Constipation and cardiovascular disease: A two-sample Mendelian randomization analysis

**DOI:** 10.3389/fcvm.2023.1080982

**Published:** 2023-02-24

**Authors:** Qichao Dong, Delong Chen, Yuxuan Zhang, Yi Xu, Longhui Yan, Jun Jiang

**Affiliations:** ^1^Department of Cardiology, Shaoxing People's Hospital (Shaoxing Hospital, Zhejiang University School of Medicine), Shaoxing, China; ^2^Department of Cardiology, Second Affiliated Hospital, College of Medicine, Zhejiang University, Hangzhou, China; ^3^Department of Cardiology, Ningbo First Hospital, Ningbo, China

**Keywords:** constipation, cardiovascular disease, Mendelian randomization, causality, atrial fibrillation

## Abstract

**Background:**

Although several observational studies have suggested positive associations between constipation and cardiovascular disease (CVD), a solid causal association has not been demonstrated. Therefore, a two-sample Mendelian randomization (MR) study was performed to investigate the causal associations between constipation and CVD.

**Methods:**

Independent genetic variants strongly associated with constipation were obtained from the FinnGen consortium. Summary-level data for CVD, including coronary artery disease (CAD), myocardial infarction (MI), heart failure (HF), atrial fibrillation (AF), stroke, and its subtypes, were collected from a few extensive genome-wide association studies (GWASs). The inverse-variance weighted methods, weighted median, and MR-Egger were used for the MR estimates. The Cochran’s Q test, MR-Egger intercept tests, MR-PRESSO, MR Steiger test, leave-one-out analyses, and funnel plot were used in the sensitivity analysis.

**Results:**

Genetically determined constipation was suggestively associated with AF risk (odds ratio (OR), 1.07; 95% confidence interval (CI), 1.01, 1.14; *p* = 0.016). Constipation and other CVD do not appear to be causally related. It was demonstrated that the results were robust through sensitivity analyses.

**Conclusion:**

This MR study demonstrated suggestive causal associations of constipation on AF, despite no associations achieving a significance value after multiple testing corrections. There was no evidence of an association between constipation and the risk of CAD, MI, HF, stroke, or stroke subtypes.

## 1. Introduction

As the primary cause of mortality and disability globally, cardiovascular disease (CVD) poses an increasingly healthy and social burden with the world’s population aging (1). In view of the severe social and clinical consequences, prompt action was required to identify risk factors of CVD for early prevention and intervention (2). Genetic or environmental factors may lead to the occurrence and progression of CVD. In addition, some studies have indicated that constipation is probably associated with CVD (3–5).

Constipation is a prevalent worldwide health issue reported daily in clinical practice (6). The prevalence of constipation among patients hospitalized for cardiovascular disease is approximately 50% (7). Several epidemiological studies have reported associations between constipation and CVD. A cohort study including 73,047 postmenopausal women showed that patients with severe constipation experienced a 23% higher risk of coronary artery disease (CAD) at a median follow-up of 6.9 years (4). In another cohort of 3,359,653 US veterans, patients with constipation experienced an 11% higher risk of CAD and a 19% higher risk of ischemic stroke (5). Meanwhile, Honkura and colleagues robustly demonstrated that constipation is significantly related to overall cardiovascular disease mortality in the general population, which was mainly related to the risk of stroke (8). Additionally, constipation also increases the risk of atrial fibrillation (AF) as well as heart failure (HF) (3).

However, it is still not fully elucidated whether the effects of constipation on CVD risk were merely biased by shared pleiotropic factors or reverse causation due to the inherent defects of conventional observational studies (9, 10). Besides, randomized controlled studies (RCTs) are time and labor-consuming to implement this topic. Recently, Mendelian randomization (MR) has been increasingly used to assess credible causal relationships between exposures and outcomes (11). Founded on the principle of the random assortment of genetic variants through meiosis, MR used genetic variations related to exposure as instrumental variables (IVs) to infer the association between risk factors (e.g., constipation) and disease outcomes (e.g., CVD) (12). Because genetic variants are randomly allocated at conception before disease onset, MR analysis could avoid confounding factors and reverse causality, further identifying causal determinants of a particular outcome (13). In the present study, a two-sample MR study was implemented to investigate the potential causality between constipation and CVD outcomes using large-scale genome-wide association study (GWAS) data.

## 2. Materials and methods

### 2.1. Study design

**Figure 1:**
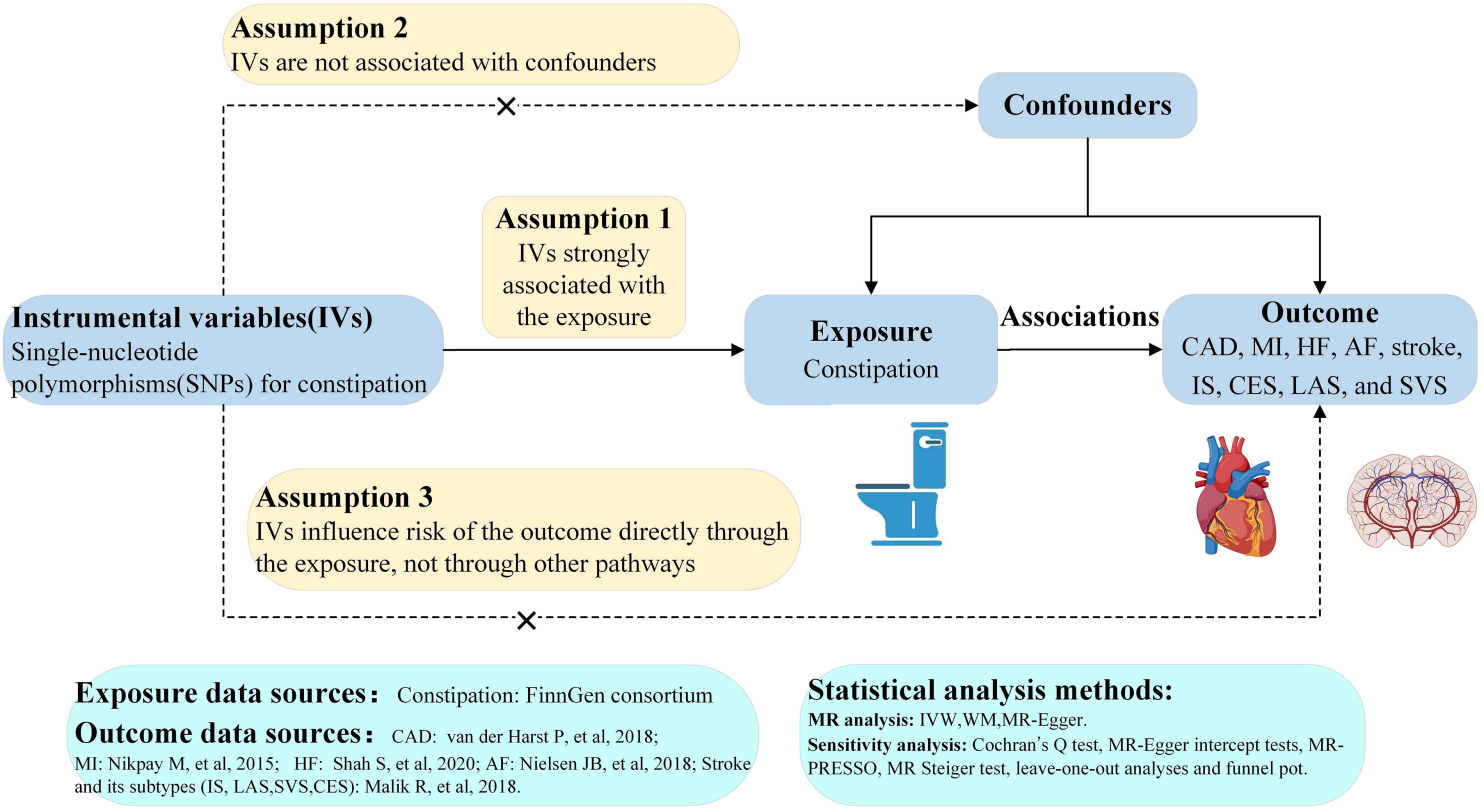
Study flow diagram. The dashed lines represent possible pleiotropic or direct causal effects between variables that might violate MR assumptions. CAD, coronary artery disease; MI, myocardial infarction; HF, heart failure; AF, atrial fibrillation; IS, ischemic stroke; CES, cardioembolic stroke; LAS, large-artery atherosclerotic stroke; SVS, small-vessel stroke; IV, instrumental variable; IVW, inverse-variance weighted; WM, weighted-median; MR, Mendelian randomization; MR-PRESSO, MR pleiotropy residual sum and outlier.

We conducted a two-sample MR study using data from the publicly available FinnGen (https://www.finngen.fi/en) and the GWAS summary data (https://gwas.mrcieu.ac.uk/). Informed consent and ethical approval were provided in the original publications and these publicly available databases. This MR analysis was founded on three critical assumptions as follows: (1) IVs must be strongly associated with constipation, (2) IVs must not be associated with confounders, and (3) IVs cannot lead to CVD unless through their effects on constipation (Figure 1) (14, 15).

### 2.2. Data sources

Summary statistics for constipation were obtained from FinnGen with a sample size of 309,154 European individuals comprising 26,919 cases and 282,235 controls (16). Genetic associations with CAD were obtained from a GWAS meta-analysis comprising 122,733 CAD cases and 424,528 controls from the CARDIoGRAMplusC4D consortium and UK Biobank (17). Summary data for myocardial infarction (MI) were also derived from the CARDIoGRAMplusC4D consortium, which comprised 171,875 participants (77% for European ancestry; 43,676 MI cases and 128,199 controls) (18). Summary-level data for HF were extracted from the HERMES consortium, including 977,323 subjects of European ancestry (47,309 HF cases and 930,014 controls) (19). Summary statistics for AF came from a large-scale GWAS meta-analysis, including 60,620 AF cases and 970,216 controls of European ancestry (20). Summary statistics for stroke were obtained from the MEGASTROKE consortium, which comprised 446,696 participants of European ancestry (40,585 stroke cases and 406,111 controls) (21). 34,217 subjects were defined as having an ischemic stroke (IS) among these stroke cases. Further, ischemic stroke was divided into three subtypes, including large-artery atherosclerotic stroke (LAS, 4373 cases), small-vessel stroke (SVS, 5386 cases), and cardioembolic stroke (CES, 7193 cases). The overlapping populations did not exist between the exposures and outcomes GWASs.

### 2.3. Selection of genetic instrumental variables and statistical power

First, we identified three single-nucleotide polymorphisms (SNPs) robustly associated with constipation (*p* < 5 × 10^−8^). Then, a more relaxed threshold (*p* < 5 × 10^−6^) was used to identify SNPs since the number of SNPs meeting genome-wide significance was limited. Second, to obtain independent SNPs, we collected SNPs at linkage disequilibrium (LD) *r*^2^ threshold at *r*^2^ < 0.001 and kb > 10,000 based on European ancestry reference data, which come from the 1,000 Genomes Project (22). Third, we calculated the *F*-statistics to test the strength of each instrument with the following formula: *F* = *R*^2^ × (*N*−2)/(1−*R*^2^) (23), where *N* represents the sample size of constipation and *R*^2^ represents the proportion of variance in constipation explained by each selected SNP (calculated by the method described previously) (24, 25). Then we selected SNPs with an *F*-statistic of more than 10 to prevent potential weak instrument bias. Fourth, we searched SNPs in PhenoScanner V2^1^ to assess whether these SNPs were associated (*p* < 1 × 10^−5^) with possible horizontal pleiotropic effects or risk factors for CVD (26). Next, we removed those SNPs with confounding traits that may influence the results. Subsequently, we extracted the remaining SNPs from the summary statistics of CAD, MI, HF, AF, stroke, and stroke subtypes. To meet the third assumption, SNPs that were significantly (*p* < 5 × 10^−6^) associated with the outcomes directly were dropped. When the specified SNP for constipation was unavailable in these outcomes data, a highly relevant SNP (*r*^2^ > 0.8) on SniPA^2^ was selected as a proxy. Without appropriate proxies available for those absent in these outcomes data, we then excluded them. Then, We excluded SNPs being palindromic based on the allele frequency after harmonizing the SNPs-exposure and SNPs-outcome. Finally, we performed MR-pleiotropy residual sum and outlier (MR-PRESSO) before MR analysis to discard any outliers with potential pleiotropy to guarantee the liability of MR estimates (27). The remaining SNPs were finally utilized as genetic instruments following the abovementioned steps. Statistical power was calculated with an online tool available at https://shiny.cnsgenomics.com/mRnd/ (28).

### 2.4. Mendelian randomization analyses

Three MR analytical methods were conducted to assess the causal effects of constipation on CVD in this study to avoid the influence of potential pleiotropic effects of genetic variants. The primary MR analysis was conducted by the random-effects inverse-variance weighted (IVW) method, which combines the Wald ratio estimates of each SNP on the outcome to gain a pooled causal estimate and provides the highest statistical power. For random-effect IVW, it permits that all the instruments are ineffective on the condition that overall horizontal pleiotropy is balanced (29). Furthermore, another two MR analyses, weighted median (WM) and MR-Egger, were implemented as complements to detect the causality. The weighted median method can generate unbiased causal estimates on the condition that at least 50% of the weight comes from valid instrumental variables (30). The MR-Egger method provides consistent estimates accounting for pleiotropy on the condition that all the instruments are invalid, although with the lowest power (31). Our MR estimates of the risk of CVD were presented as odds ratio (OR, 95% confidence interval [CI]) per-1-log unit increase in the risk of constipation. A two-sided value of *p* < 0.05 were deemed as suggestive significance and associations with *p*-values <0.0056 (Bonferroni correction *p* = 0.05/9 outcomes) were considered statistically significant.

### 2.5. Sensitivity analysis

Sensitivity analysis was conducted to detect the existence of horizontal pleiotropy, which violated the main MR assumptions. Thus, we perform Cochran’s Q test, MR-Egger intercept tests, MR-PRESSO, MR Steiger test, leave-one-out (LOO) analyses, and funnel plot to examine the presence of pleiotropy to evaluate the robustness of the results. Specifically, the Cochran *Q* test was applied to evaluate the heterogeneity, which was detected if the *p* value was less than 0.05. Horizontal pleiotropy was appraised by estimating the intercept term derived from MR-Egger regression, indicating potential bias with the intercept term difference from 0 (31). MR Steiger test was applied to estimate the potential reverse causal association between CVD and constipation (32). The LOO analysis was performed to detect any pleiotropy driven by a single SNP.

All these MR analyses were performed using the TwoSampleMR package (version 0.5.6) in R Version 4.2.1.

## 3. Results

### 3.1. Genetic instruments selected in Mendelian randomization

In this study, we obtained 20 SNPs associated with constipation, which met the universally accepted genomewide significance threshold (*p* < 5 × 10^−6^, *r*^2^ < 0.001, kb = 10,000) for exposure (Supplementary Table S1). One SNP (rs2130630) in constipation was removed to eliminate confounding factors associated with body mass index. Furthermore, estimates of the F-statistic suggested that no weak instrument was employed in our MR analysis (all F-statistics >10) (Supplementary Table S1). No relevant proxy SNPs were identified to replace the small number of SNPs (2–4) absent in different CVD GWASs data. After removing outliers identified by MR-PRESSO and excluding ambiguous and palindromic SNPs through harmonizing processes, the remaining SNPs were selected as instrumental variables. Details of instrumental variables of each CVD were exhibited in Supplementary Tables S2–S10. In the present study, given a type I error of 5% and a statistical power of 0.80, the minimum detectable ORs for the 9 CVDs ranged from 1.31 to 2.41.

### 3.2. Causal effects of constipation on CVD

**Figure 2:**
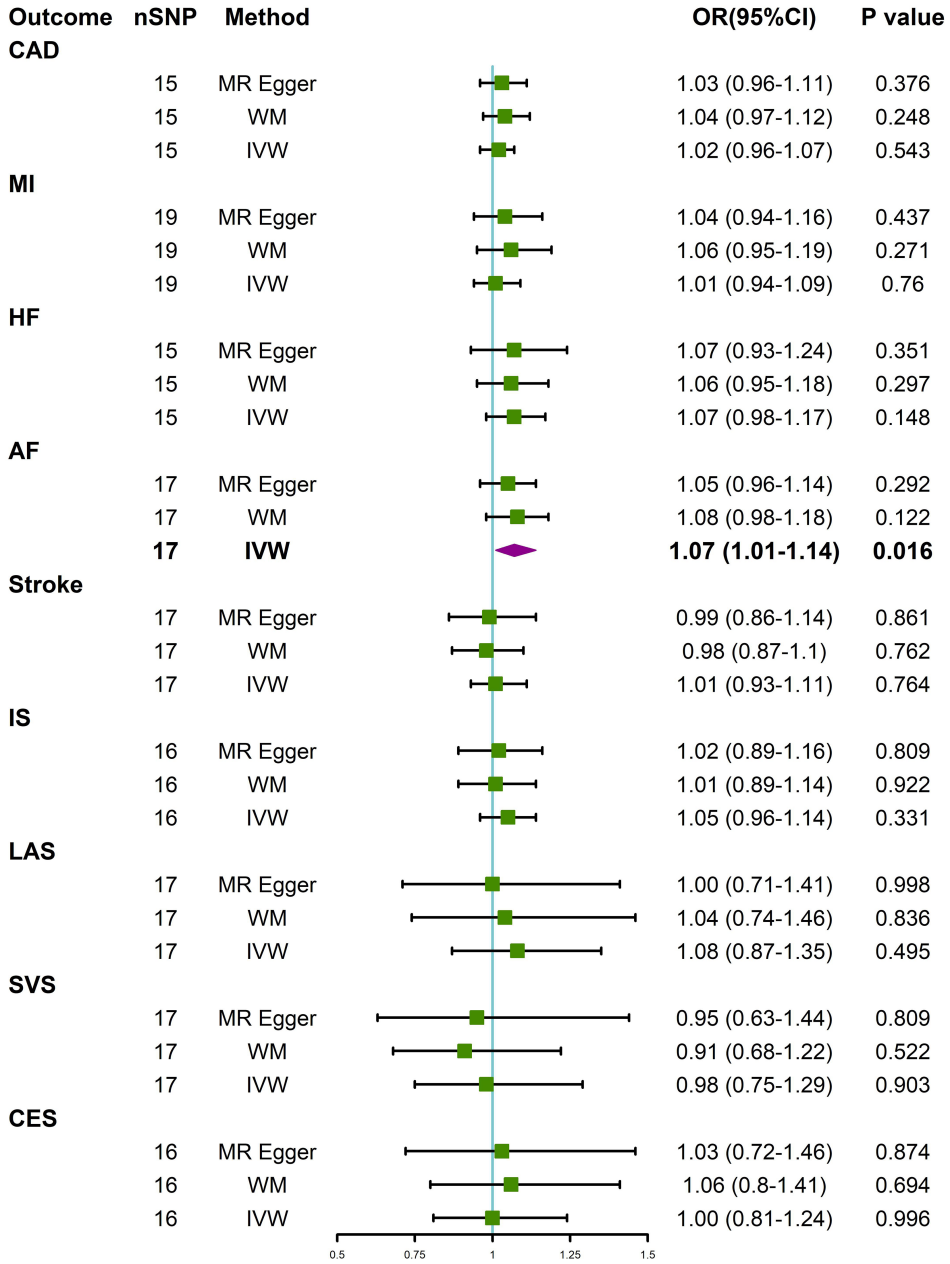
Causal effects for constipation on CVD. MR-Egger, weighted median (WM), and inverse-variance weighted (IVW) estimates of Mendelian randomization (MR) are summarised. CI, confidence interval; nSNP, number of single nucleotide polymorphism; OR, odds ratio. See Figure 1 for other abbreviations.

IVW analysis showed that constipation was suggestively associated with the risk of AF (OR = 1.07, 95% CI 1.01–1.14; *p* = 0.016). The results from WM and MR-Egger indicated a nonsignificant but consistent direction (Figure 2). There was no significant or suggestive association between genetic liability to constipation and the risk of CAD, MI, HF, stroke, or stroke subtypes (all *p* > 0.05). The results were consistent with IVW, WM, and MR-Egger (Figure 2). However, we may not have reached sufficient statistical power to detect such weak associations.

### 3.3. Sensitivity analyses

To evaluate the robustness of the results, several sensitivity analyses, consisting of Cochran’s *Q* test, MR-PRESSO global test, MR Steiger test, and MR Egger intercept test, were conducted (Table 1). All *p* values were > 0.05 in the MR-PRESSO global tests and the MR-Egger intercept tests, manifesting that no horizontal pleiotropy existed across the analyses. MR Steiger test identified no evidence of reverse causality, and the causal direction was reliable. Nevertheless, heterogeneity was detected in Cochran’s *Q* test analysis between constipation and HF (*Q* = 24.63, *p* = 0.04), constipation and SVS (*Q* = 27.47, *p* = 0.04). However, the detected heterogeneity in certain results did not invalidate the MR estimates because the random-effect IVW used in this study could balance the pooled heterogeneity. Aside from that, the MR-Egger intercepts did not reveal any pleiotropy, which suggests that MR estimates were not biased by heterogeneity (Supplementary Figures S1–S9). Other analyses did not find any heterogeneity. Furthermore, after deleting 1 SNP at a time from the LOO analysis, the risk estimates did not change much, proving that no specific SNP was critical for the causal association (Supplementary Figures S10–S18). Moreover, as shown by the funnel plot, the effect size variation around the point estimate was symmetrical, meaning that horizontal pleiotropy was not apparent (Supplementary Figures S1–S9).

**Table 1:** Sensitivity analysis of the causal association between constipation and the risk of CVD.

Outcome	Cochran *Q* test		MR-PRESSO	MR-Egger		MR steiger test
	*Q*_value	*p*_value	*p*_value	Intercept	*p*_value	*p*_value
CAD	8.15	0.88	0.76	−0.003	0.50	9.05E-40
MI	10.94	0.90	0.81	−0.005	0.42	1.93E-41
HF	24.63	0.04	0.07	−0.0003	0.96	2.92E-45
AF	14.48	0.56	0.60	0.003	0.48	1.96E-55
Stroke	19.93	0.22	0.25	0.003	0.63	3.40E-35
IS	15.19	0.44	0.47	0.003	0.61	4.02E-34
LAS	16.08	0.45	0.51	0.010	0.56	1.02E-15
SVS	27.47	0.04	0.06	0.005	0.83	1.01E-16
CES	20.58	0.15	0.19	−0.003	0.83	1.57E-18

See Figure 1 for abbreviations.

## 4. Discussion

Constipation incidence varies from 3 to 79% in diverse adult groups, depending on age, gender, and definition of constipation (33, 34). Though constipation has imposed an immense social and economic burden, limited attention was paid to it in the medical field, which causes its genesis and physiopathology to be poorly elucidated (35). Consequently, there is little information about the potential relationship between constipation and cardiovascular risk. Though a few researchers have examined the relationship between constipation and CVD, the majority of them only concentrate on stroke and CAD (4, 5, 36, 37). Meanwhile, the results from previous works of literature are confined to observational correlations, and reverse causality may be unavoidable.

Utilizing the available large-scale GWAS data, we adopted MR which is a time- and labor-saving way to examine the association between constipation and the risk of nine CVDs. Intriguingly, genetically determined constipation is suggestively associated with enhanced AF risk was revealed. No clear pattern of associations of genetically determined constipation with the risk of CAD, MI, HF, stroke, or stroke subtypes was found. Five sensitivity tests revealed that causal effects were not caused by outliers, horizontal pleiotropy, or reverse causality. To the best of our knowledge, this is the first MR study to estimate the causal association between constipation and CVDs.

Our finding that there is a suggestive causal link between constipation and the risk of AF concurs with a Danish population-based matched cohort study that found a 27% higher risk of AF in those with constipation (3). However, the mechanisms underlying this association are unidentified. Gut microbiota imbalance caused by constipation may be one of the possible explanations for the causality. Studies have revealed that the gut microbiota of those with constipation differs from those of healthy individuals (38, 39). Unbalanced gut microbiota may cause the intestinal mucosal barrier to disrupt, resulting in inflammation, cytokine release, and immune suppression (40–42). On the flip side, gut microbiota dysbiosis may be made worse by higher levels of inflammation, leading to aberrant bowel function and the ensuing major chronic illnesses, such as AF (43). Interestingly, Zhang et al. provided solid evidence that gut microbiota dysbiosis directly contributes to the pathophysiology of AF by raising the levels of circulating LPS and glucose and activating the atrial NLRP3 inflammasome (44). Besides, increased blood pressure linked to gut microbiota dysbiosis may also lead to AF (45). Additionally, oxidative stress and constipation-induced anxiety may be another link between constipation and AF (7, 46). Nevertheless, the causal role of constipation in AF needs to be interpreted cautiously and future study is warranted to investigate the potential mechanisms.

In the last decade, several observational studies that explored the relationship between chronic constipation and CAD, MI, HF, stroke, and its subtypes yielded contradictory results (3–5, 8, 36, 37). Elena et al. conducted a cohort study in postmenopausal women, revealing that only the severe constipation group was associated with an increased risk of cardiovascular events, including CAD, MI, and stroke and its subtypes (4). In patients from US veterans, Keiichi et al. demonstrated that patients with constipation and patients using laxatives experienced a similarly higher risk of CAD and ischemic stroke (5). However, Yasuhiko et al. reported that the risk of constipation on CAD and stroke would no longer be statistically significant after adjusting for potential confounding variables (36). Our MR analysis does not provide evidence of the causal effects of constipation on CAD, MI, HF, stroke, or stroke subtypes, suggesting that associations observed clinically are likely to be biased. Thus, further studies are needed to clarify whether there are driving factors that account for bias or confounding in previous observational studies.

The IVW method generally has significantly greater statistical power than the other MR approaches, particularly MR-Egger (47). Therefore, in most cases, IVW was used as the primary method for identifying potentially significant outcomes. Other MR methods and sensitivity analyses were conducted to ensure that IVW estimates were robust. Our study also used the consistent beta direction requirement in all MR approaches, as is the case for most MR analyses (48, 49).

This study possesses several strengths. The major strength is the MR design we used for evaluating independent causal effects of constipation on multiple CVD outcomes without interference from reverse causality or residual confounding. Additionally, we used the most significant GWAS to reduce the “winner’s curse,” even though some causal estimates were relatively small. Another advantage is the magnitude of the sample size, which allowed us to perform an adequately powered MR analysis.

The current study also has several drawbacks. Firstly, even though we used the biggest GWAS on constipation, only a small number of SNPs conform to genome-wide significance, which can result in the use of weak genetic instruments. To remedy this, we eased the statistical threshold (*p* < 5 × 10^−6^) to provide additional SNPs whose F-statistics are all above 10. When bigger GWAS numbers become available, further study will be needed to corroborate our findings. Secondly, it is challenging to completely rule out pleiotropy since the biological functions of the chosen SNPs are still unclear. However, given that we cannot discover any horizontal pleiotropy in our research, it is reassuring that the causal effect estimates were robust through various MR models and sensitivity analyses depending on different assumptions. Thirdly, the statistical power of this study may be insufficient since only 0.1% of the variance in constipation was explained by IVs. Therefore, we should be cautious with interpreting the negative results; the null association might be due to a lack of power. Fourthly, since the GWAS used in our study derives from participants of European ancestry, the findings cannot be generalized to other ethnic groups. Due to these limitations, future studies are needed to confirm the causality and investigate potential mechanisms, which is compulsory for making pertinent clinical suggestions.

## 5. Conclusion

This MR study demonstrated suggestive causal associations of constipation on AF, despite no associations achieving a significance value after multiple testing corrections. There was no evidence of an association between constipation and the risk of CAD, MI, HF, stroke, or stroke subtypes.

## Data availability statement

The original contributions presented in the study are included in the article/Supplementary material, further inquiries can be directed to the corresponding author.

## Ethics statement

Ethical approval and written informed consent were provided in the original publications and these publicly available databases.

## Author contributions

QD and JJ designed this study and drafted the manuscript. QD, DC, YZ, YX, LY, and JJ contributed to the data acquisition and data analysis. All authors contributed to the article and approved the submitted version.

## Funding

This study was supported by the National Natural Science Foundation of China (grant 82170332).

## Conflict of interest

The authors declare that the research was conducted in the absence of any commercial or financial relationships that could be construed as a potential conflict of interest.

## Publisher’s note

All claims expressed in this article are solely those of the authors and do not necessarily represent those of their affiliated organizations, or those of the publisher, the editors and the reviewers. Any product that may be evaluated in this article, or claim that may be made by its manufacturer, is not guaranteed or endorsed by the publisher.

## Footnotes

^1^ http://www.phenoscanner.medschl.cam.ac.uk/

^2^ https://snipa.helmholtz-muenchen.de/snipa3/index.php
