# Supplementary material for: Constipation and cardiovascular disease: A two-sample Mendelian randomization analysis
Source: Front Cardiovasc Med. 2023 Feb 24;10:1080982. doi: 10.3389/fcvm.2023.1080982 (PMC9998987; doi:10.3389/fcvm.2023.1080982)
Supplement: Supplementary file 2 [file Data_Sheet_2.docx]

Supplementary Figures


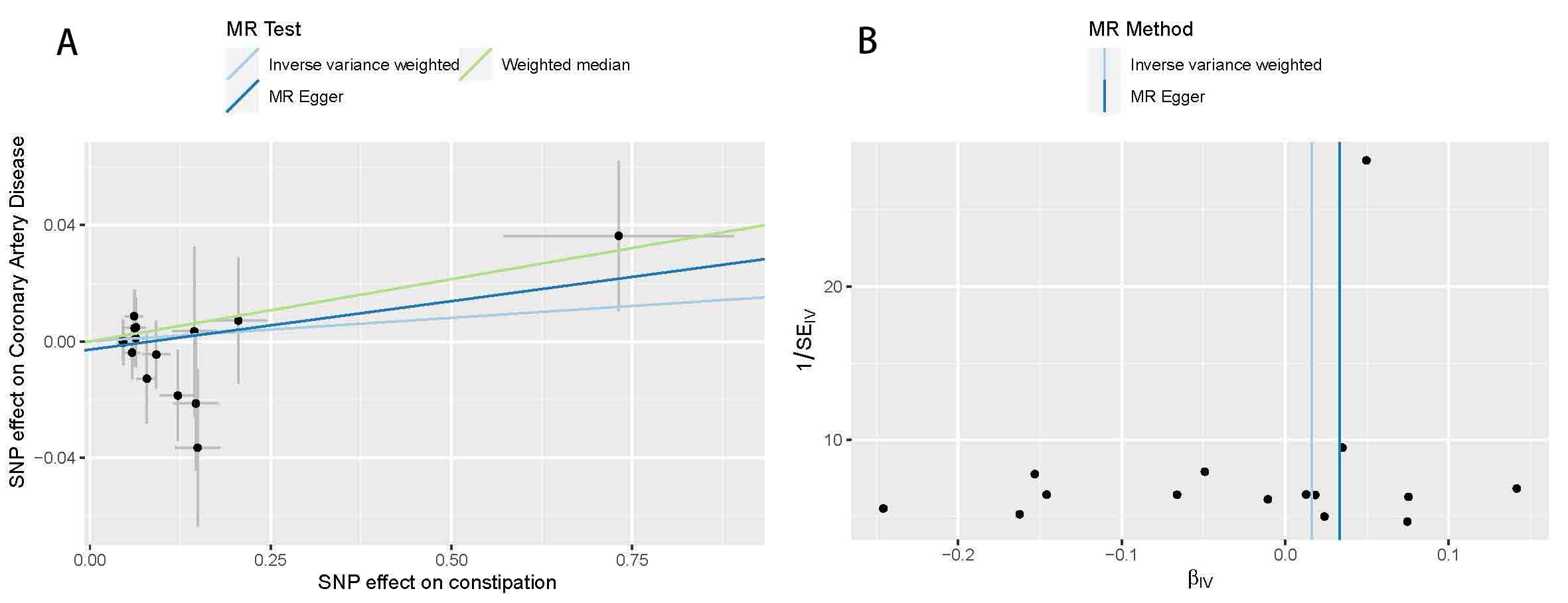


**Supplementary Figure 1.** Scatter plot (A) and funnel plot (B) from genetically predicted constipation on coronary artery disease.


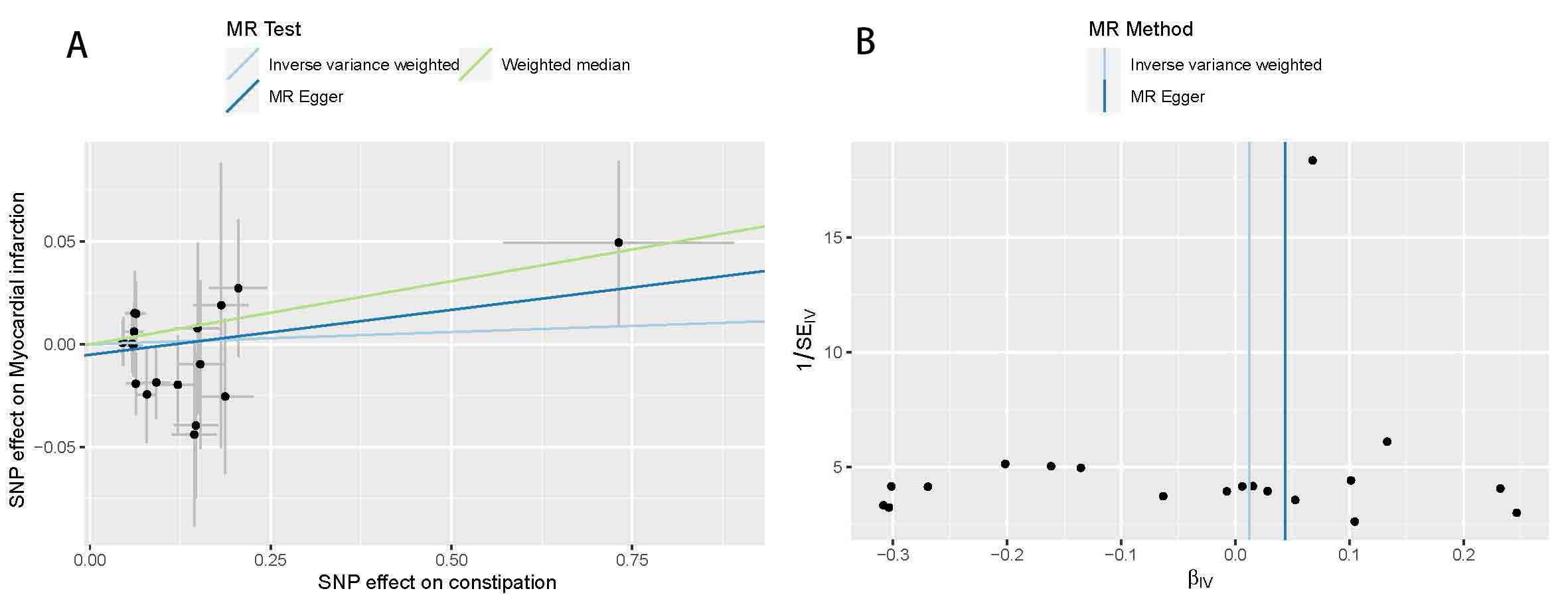


**Supplementary Figure 2.** Scatter plot (A) and funnel plot (B) from genetically predicted constipation on myocardial infarction.


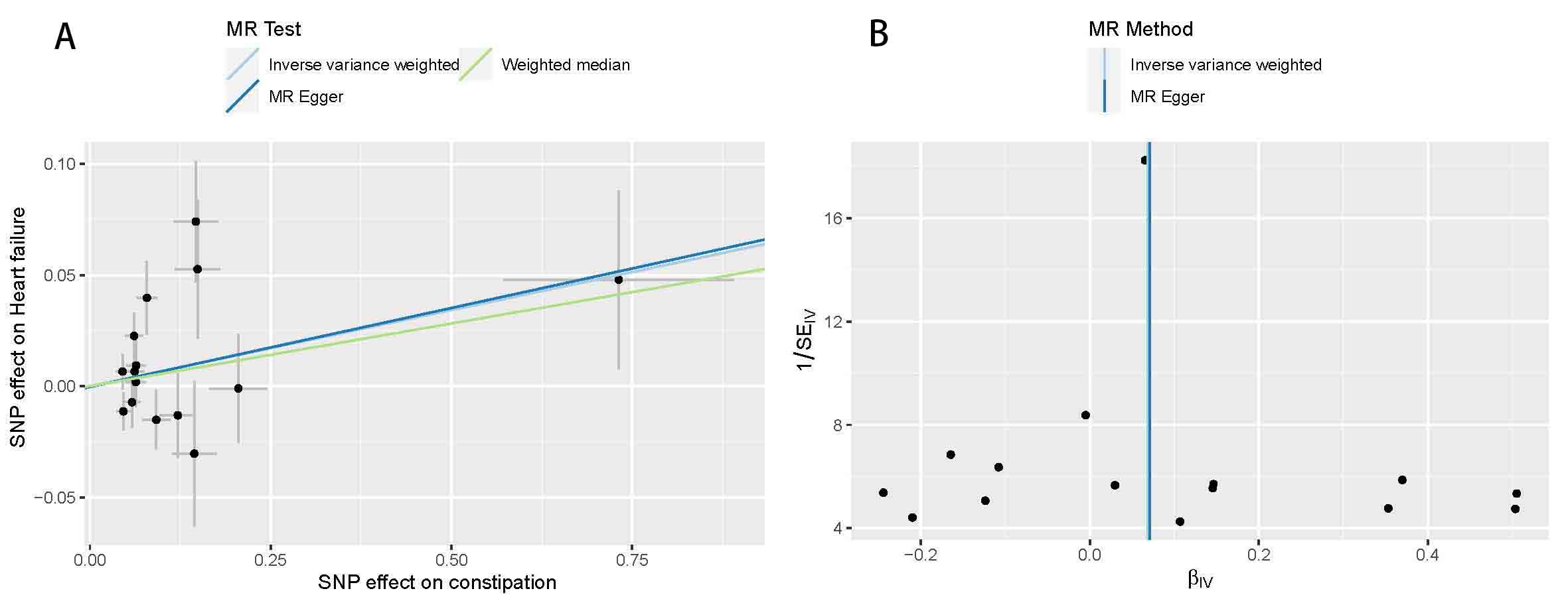


**Supplementary Figure 3**. Scatter plot (A) and funnel plot (B) from genetically predicted constipation on heart failure.


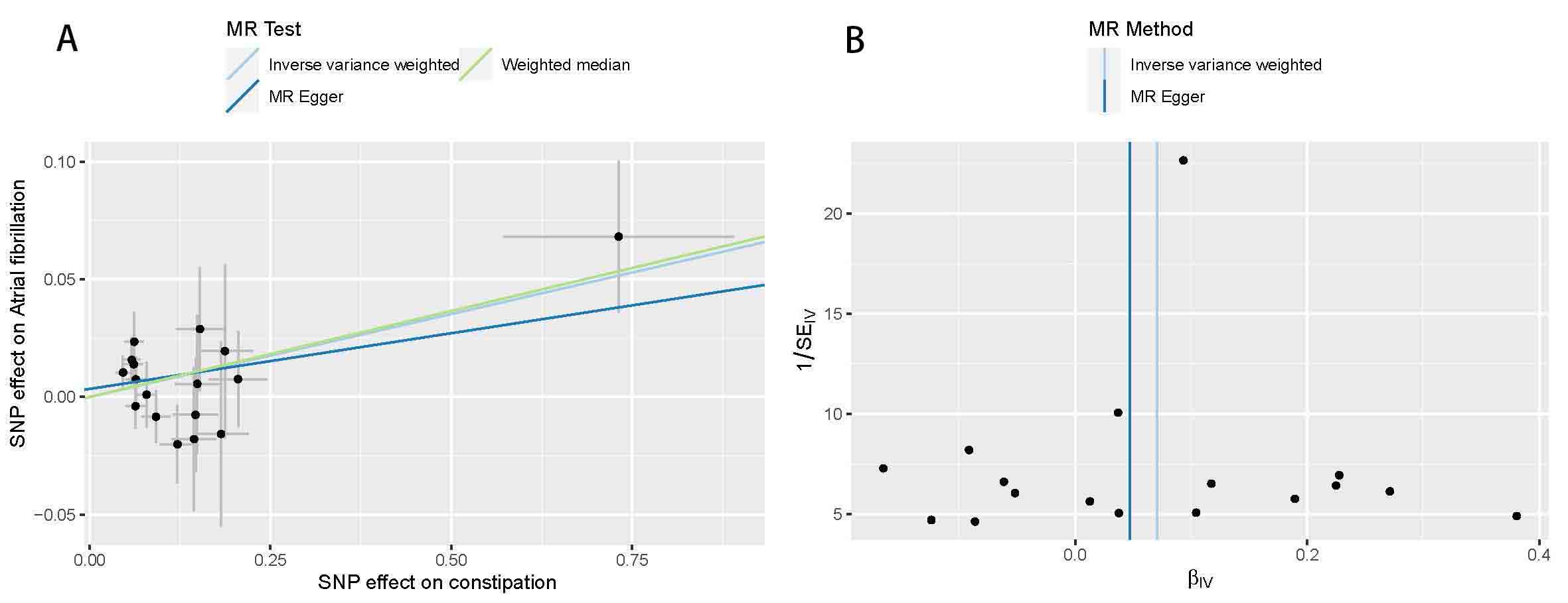


**Supplementary Figure 4.** Scatter plot (A) and funnel plot (B) from genetically predicted constipation on atrial fibrillation.


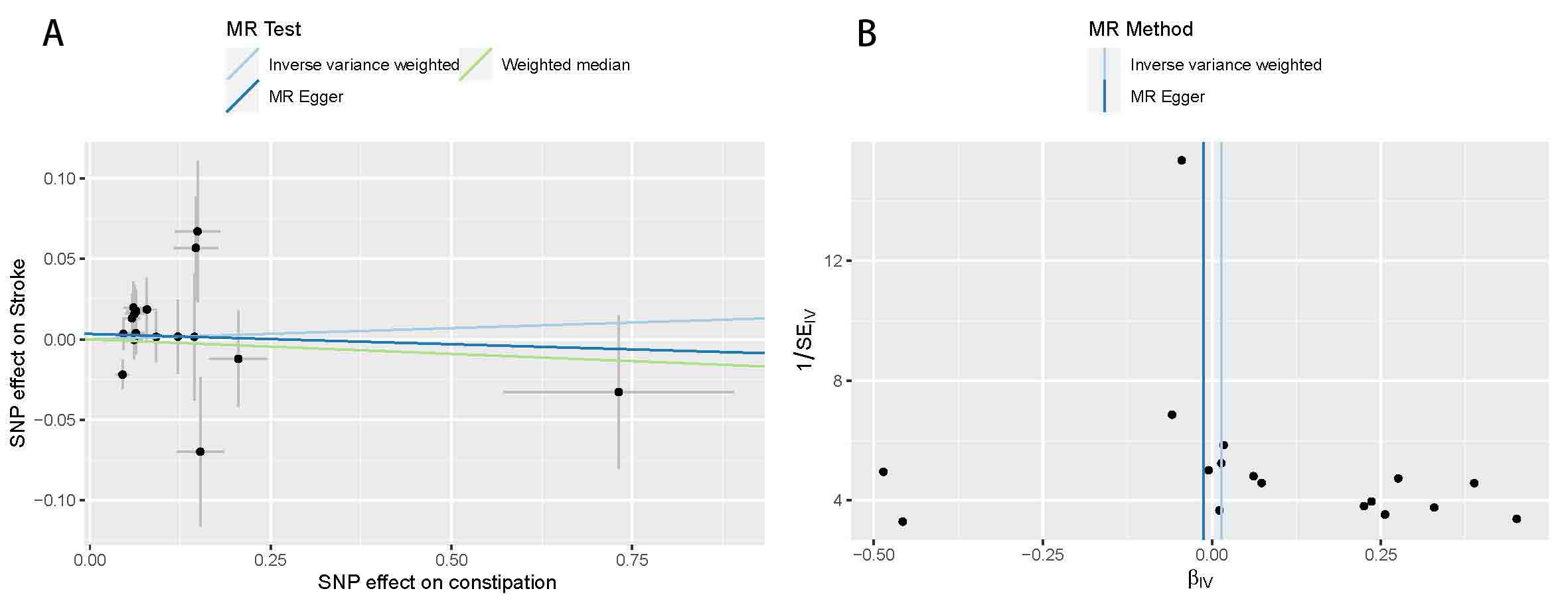


**Supplementary Figure 5.** Scatter plot (A) and funnel plot (B) from genetically predicted constipation on stroke.


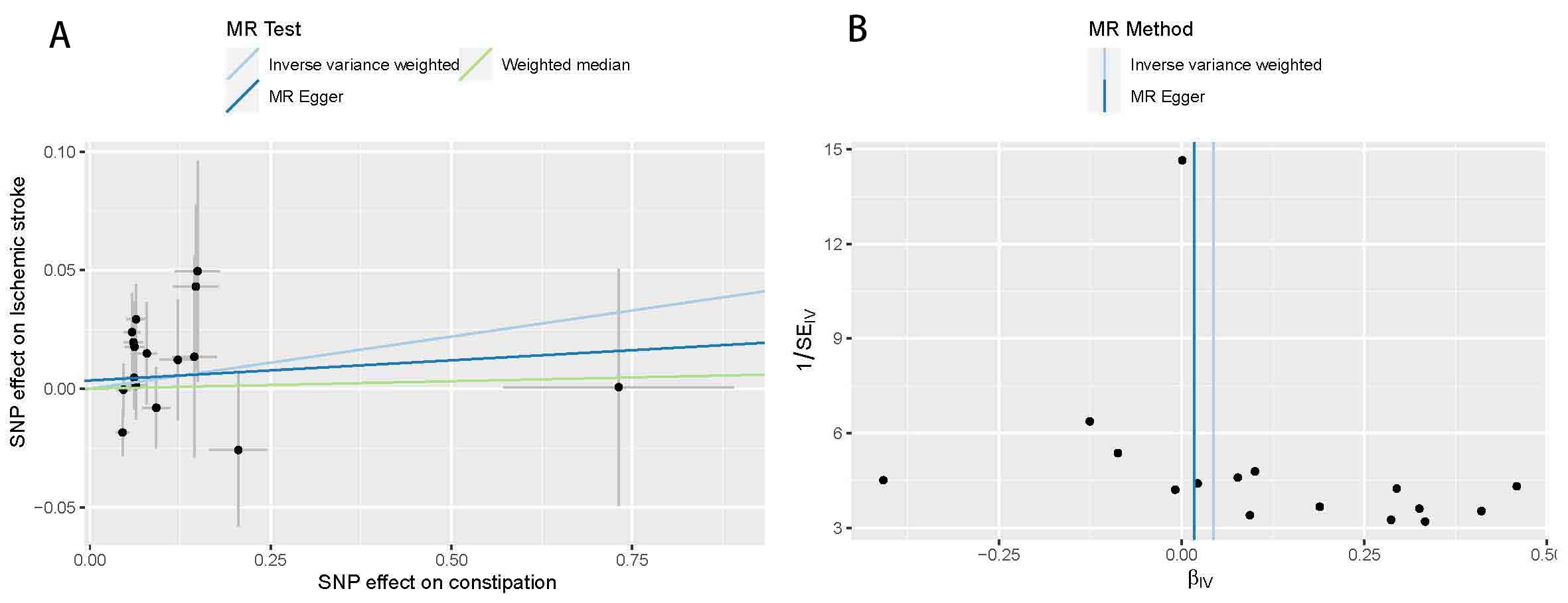


**Supplementary Figure 6.** Scatter plot (A) and funnel plot (B) from genetically predicted constipation on ischemic stroke.


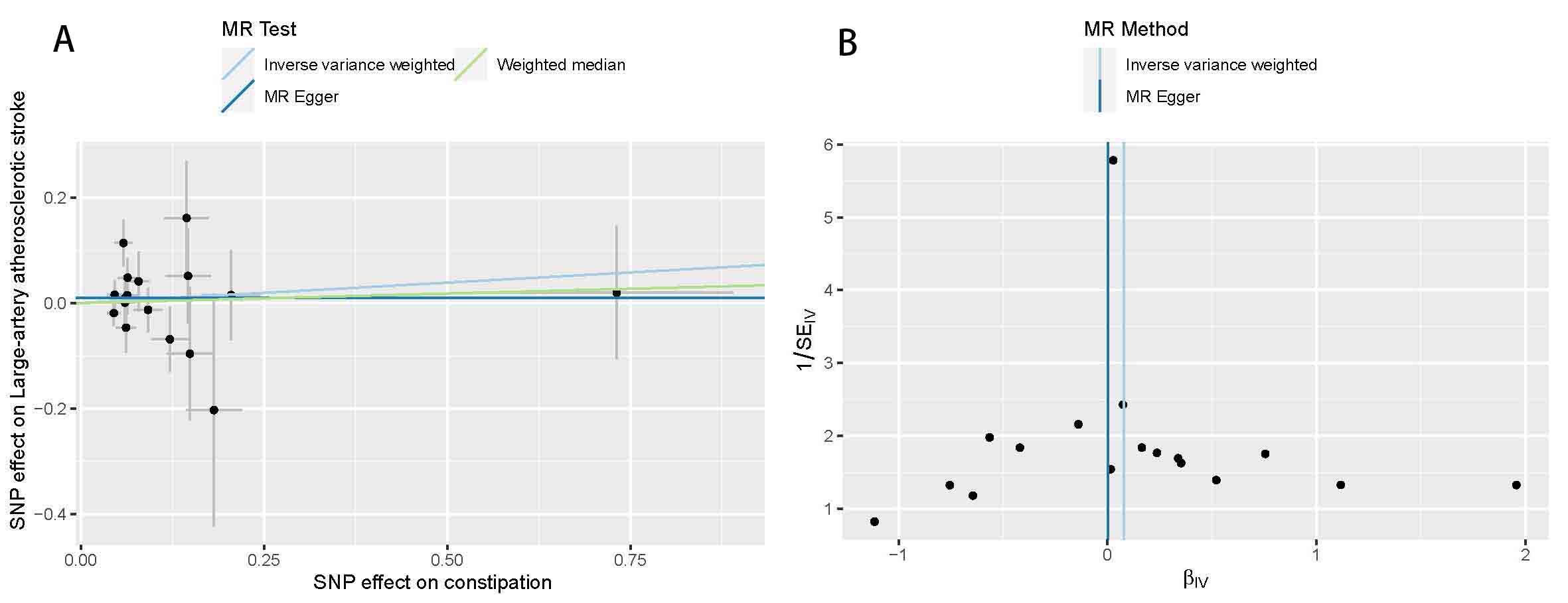


**Supplementary Figure 7.** Scatter plot (A) and funnel plot (B) from genetically predicted constipation on large-artery atherosclerotic stroke.


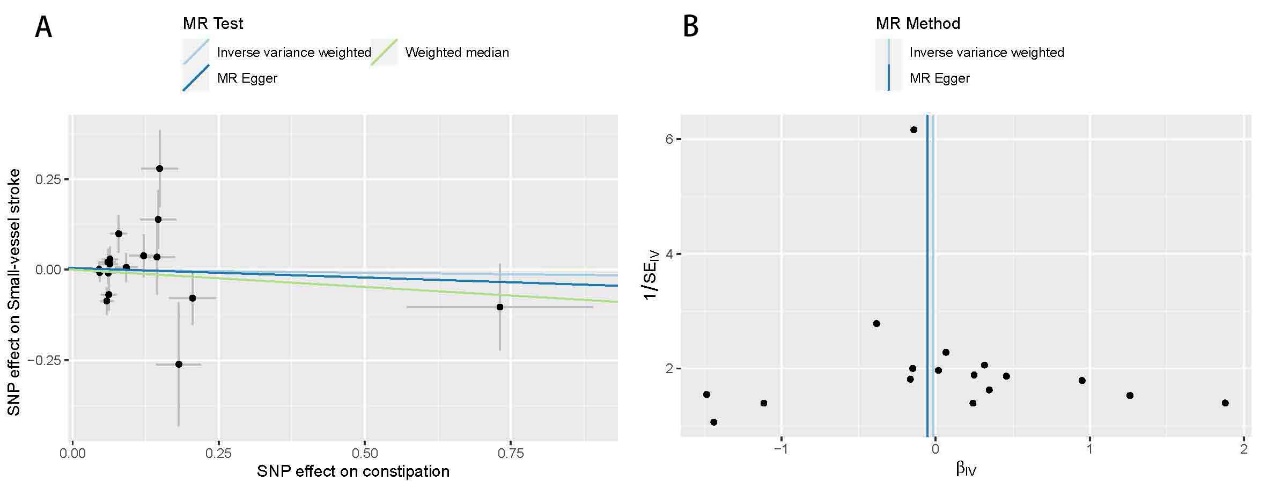


**Supplementary Figure 8.** Scatter plot (A) and funnel plot (B) from genetically predicted constipation on small-vessel stroke.


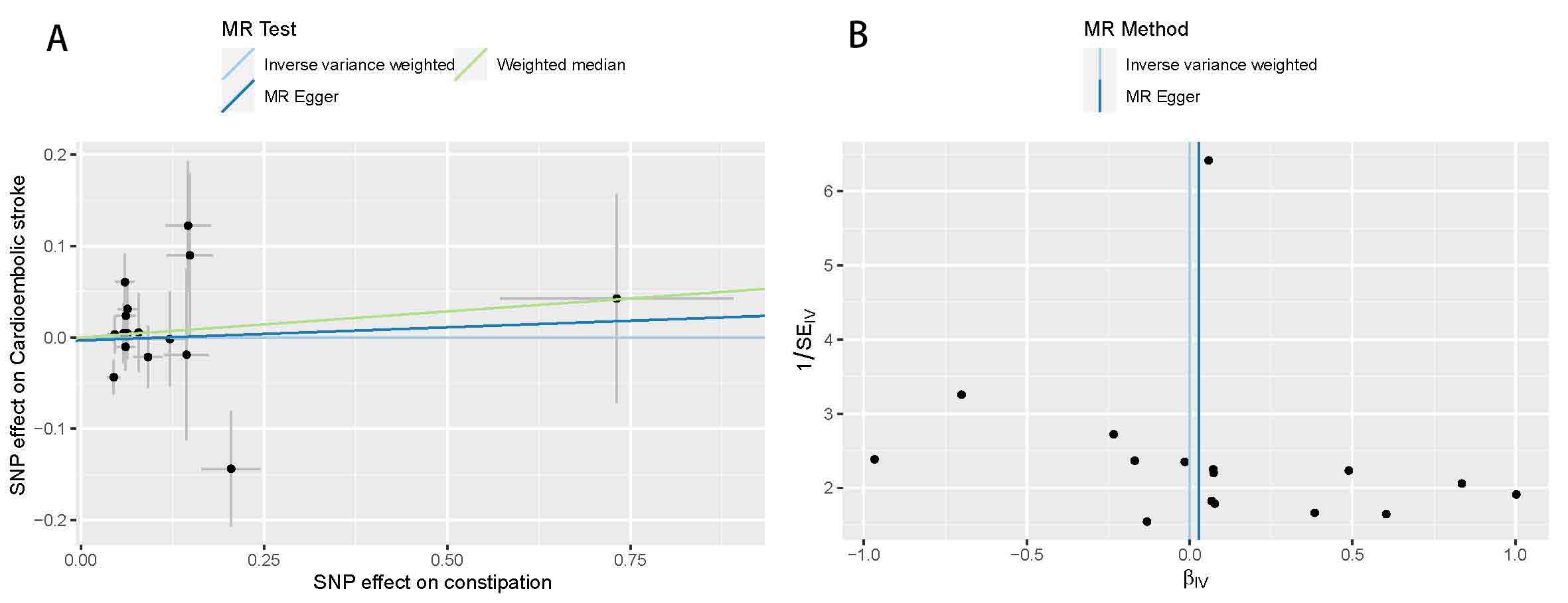


**Supplementary Figure 9.** Scatter plot (A) and funnel plot (B) from genetically predicted constipation on cardioembolic stroke.


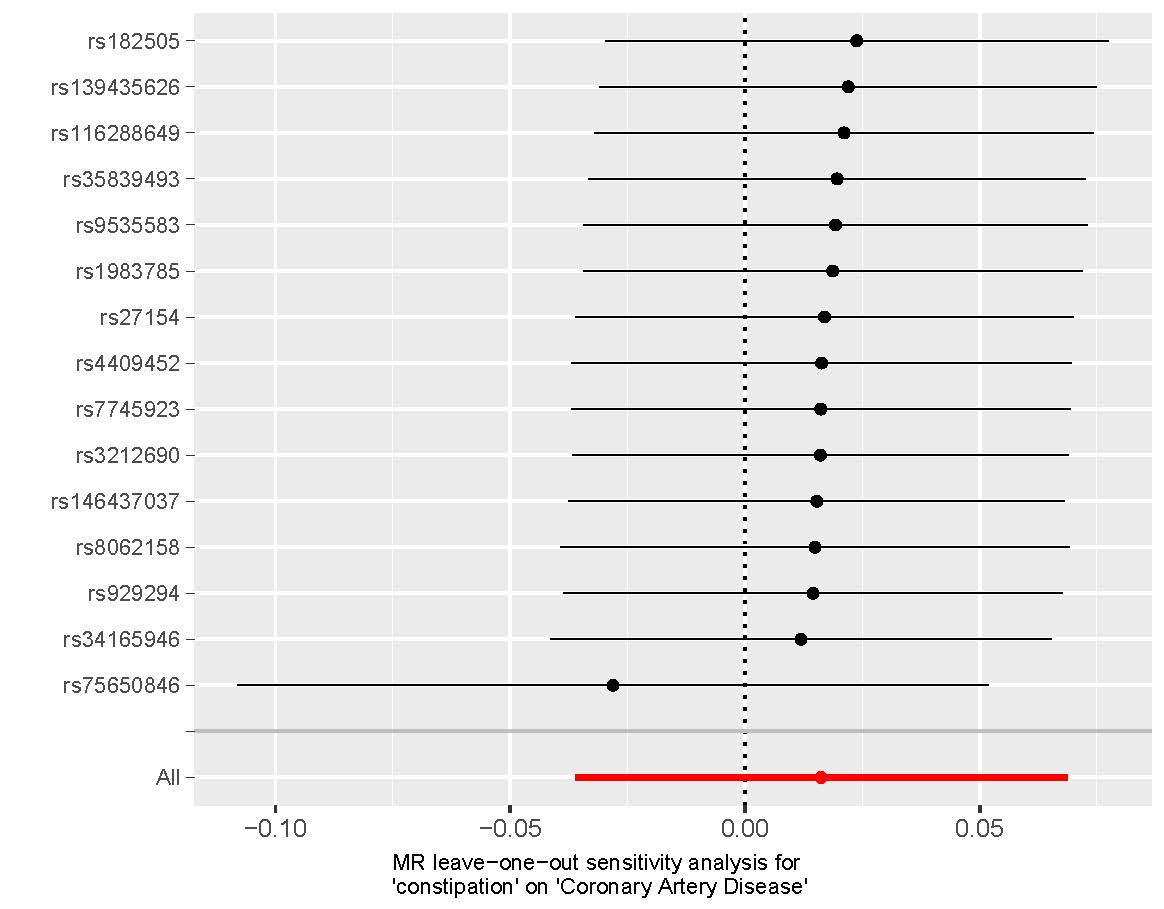


**Supplementary Figure 10.** Leave-one-out analysis for constipation on coronary artery disease.


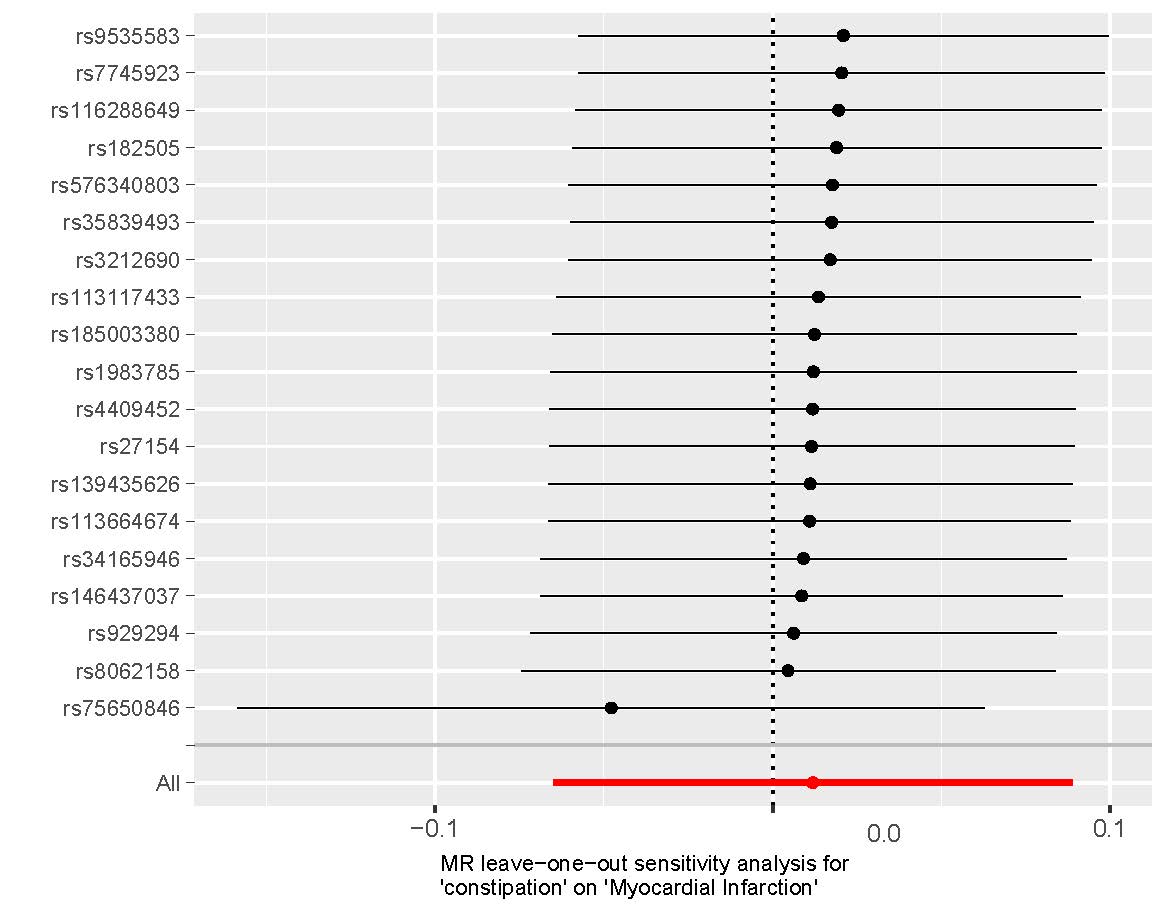


**Supplementary Figure 11.** Leave-one-out analysis for constipation on myocardial infarction.


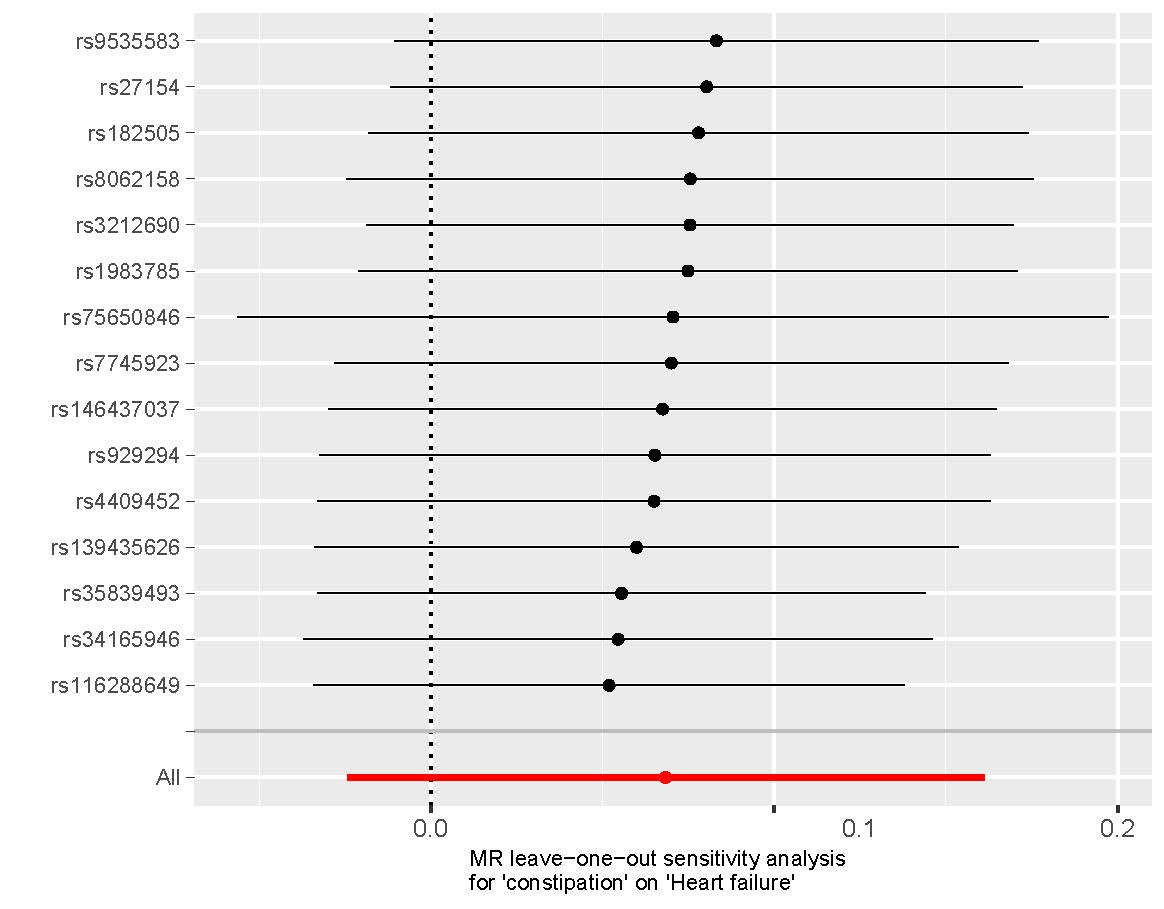


**Supplementary Figure 12.** Leave-one-out analysis for constipation on heart failure.


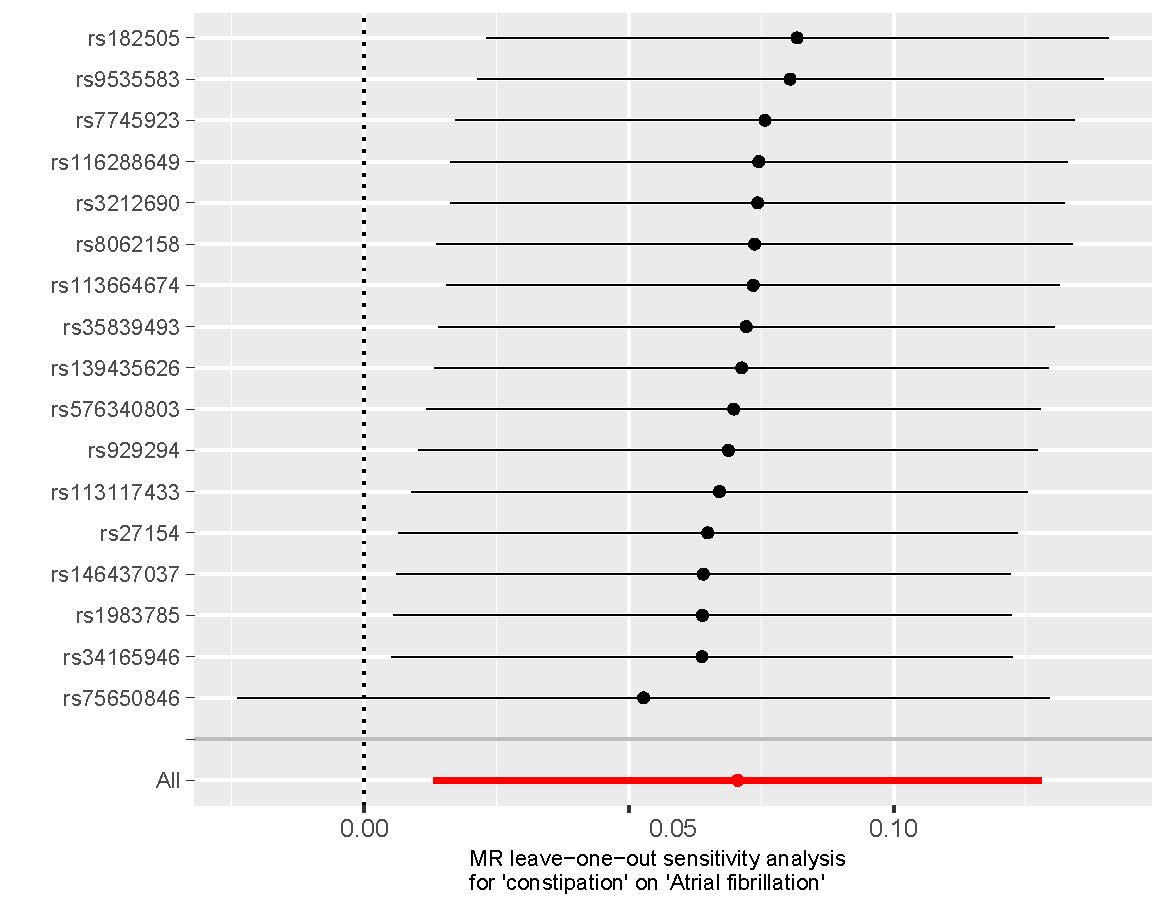


**Supplementary Figure 13.** Leave-one-out analysis for constipation on atrial fibrillation.


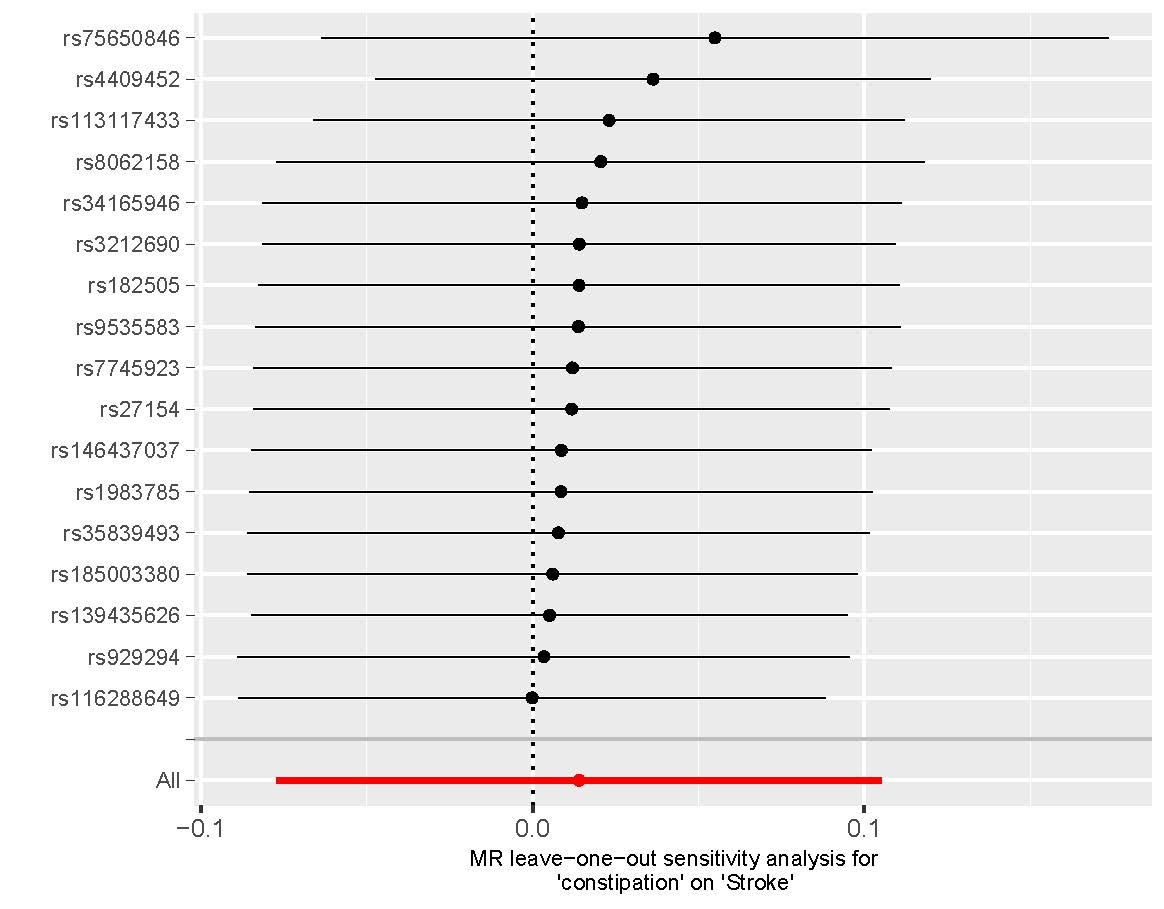


**Supplementary Figure 14.** Leave-one-out analysis for constipation on stroke.


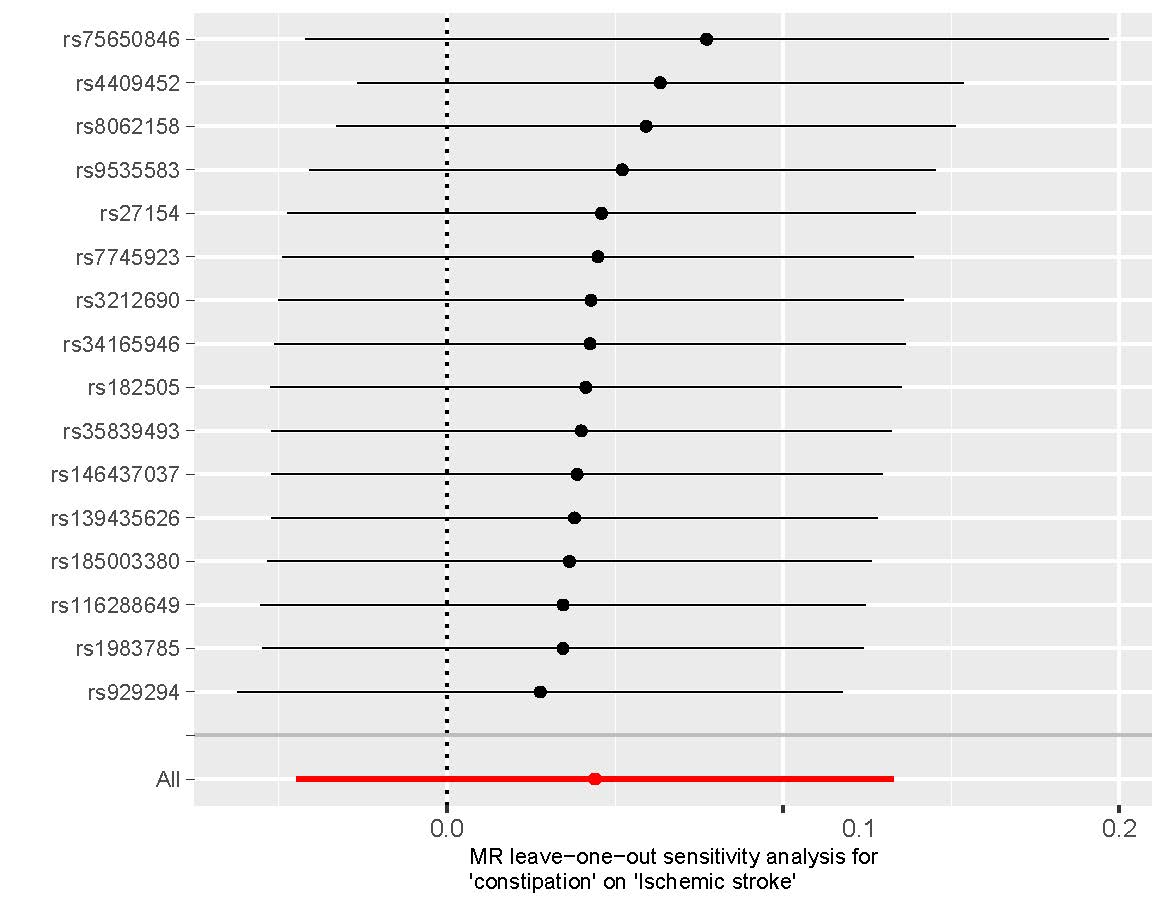


**Supplementary Figure 15.** Leave-one-out analysis for constipation on ischemic stroke.


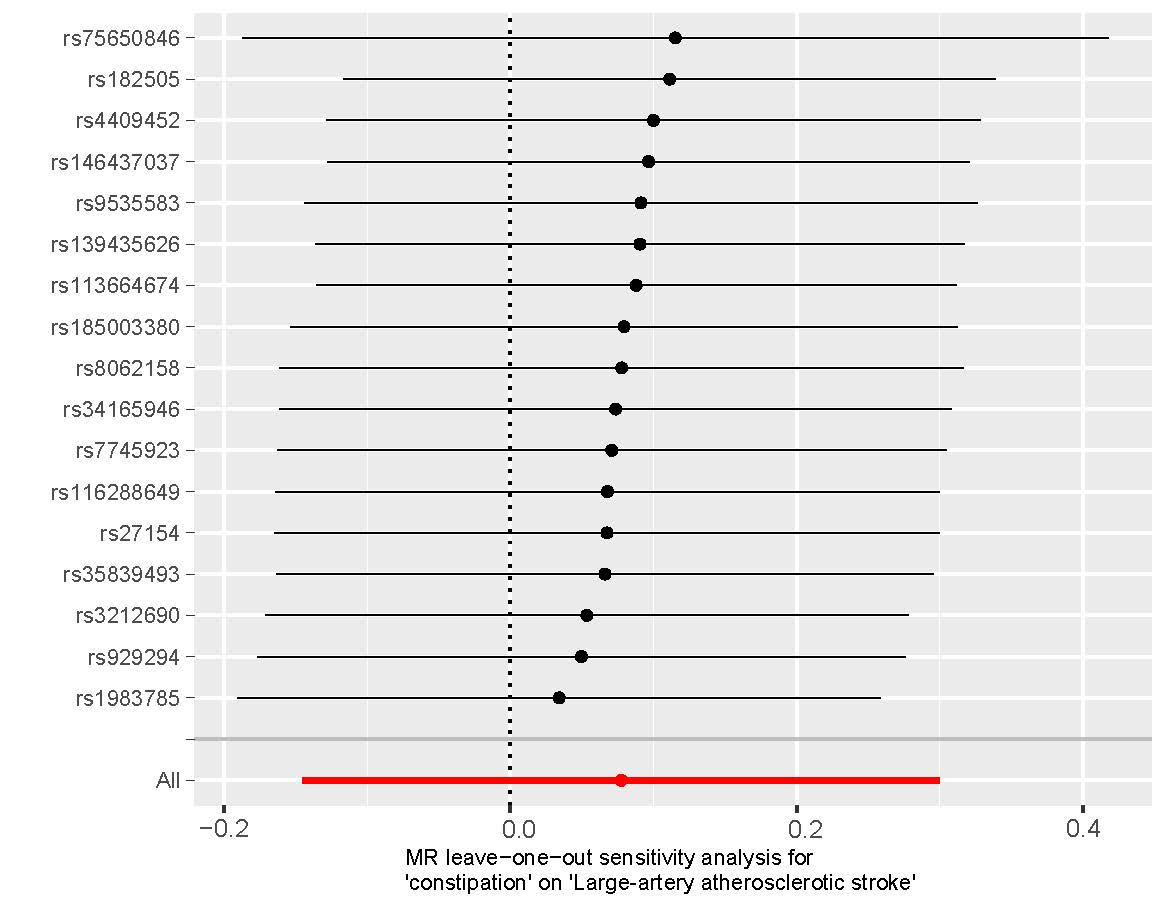


**Supplementary Figure 16.** Leave-one-out analysis for constipation on large-artery atherosclerotic stroke.


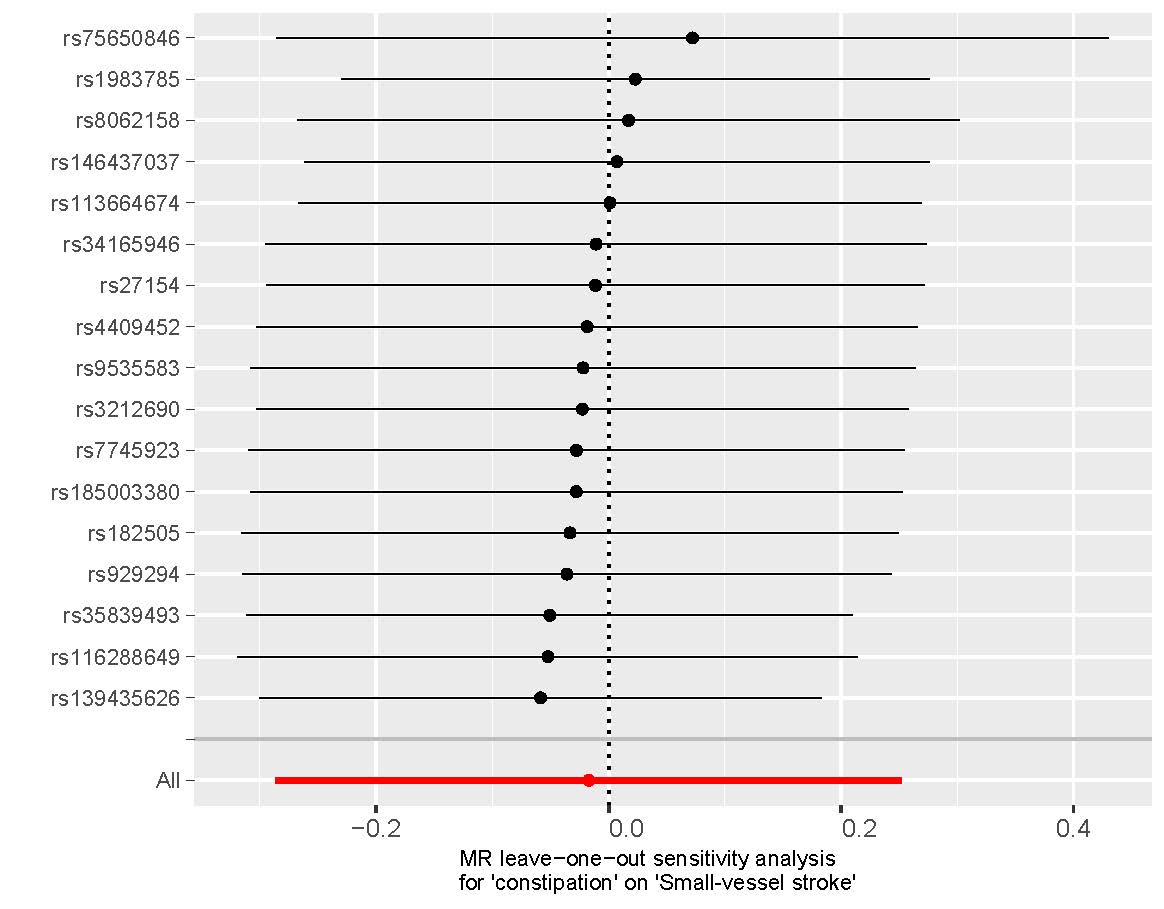


**Supplementary Figure 17.** Leave-one-out analysis for constipation on small-vessel stroke.


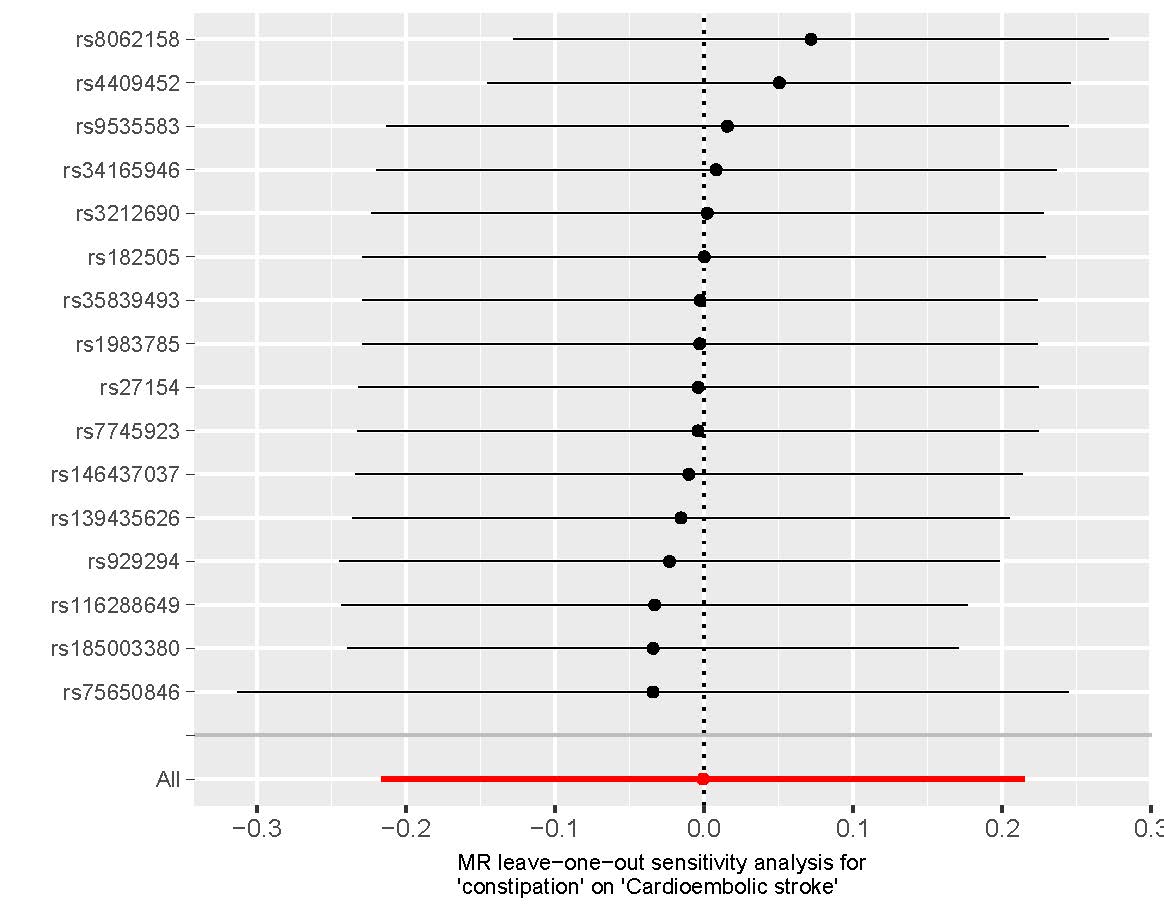


**Supplementary Figure 18.** Leave-one-out analysis for constipation on cardioembolic stroke.
